# Medical Students in Paris

**Published:** 1896-04

**Authors:** 


### Medical Students in Paris.

It is not only our own country that suffers from what might be termed a plethora of professional men. The Faculty of Medicine at Paris, alarmed at the overcrowding of the clinics and the courses of practical instruction in surgery, anatomy, pathology, etc., has adopted new regulations, both as regards students of native birth, and particularly foreigners. The standard for native students has been raised, the baccalaureate in letters alone being no longer sufficient in order to matriculate in the medical school, an extra year in scientific work being required in the future. In the case of foreigners two baccalaureates are required—the A.B. and the B.S.—and as far as possible students from other lands will be directed to the provincial schools, at least for the first two or three years of their medical studies. These regulations apply only to those that matriculate as candidates for a degree. Paris will undoubtedly prove as hospitable to those that go there for special work only, as it has heretofore been. But even if all foreign students should find the *entree* to medical work in that beautiful capital more difficult than it formerly was, yet we cannot blame the faculty for its move, which is really one in the right direction. Moreover, "charity begins at home," and if the French medical student has been prevented from enjoying the full advantages of the opportunities for medical instruction offered at Paris, through the influx of strangers, the faculty is but doing its duty to restrict such an abuse. The number of medical students in France has nearly doubled since 1885. And now, out of over 5,000 students nearly one-quarter are foreigners. As the degree given them confers the same privileges upon them as upon native-born physicians it seems but just that the foreign-born should be placed on the same footing as regards preparation and time required for the degree.—*Pacific Medical Journal.*

THE medical cuckoo is the American physician that rushes to the public to proclaim to the world the "infallible" value of every sensational fad sprung in a foreign country. When will American physicians (and American girls) overcome their sensational, mawkish yearning for exotic oddities? Let's be original; we have the brains in America.—*North American Medical Review.*

"WE have been emancipated from the trammels of theory and superstition, and the medicine of to-day is largely based upon exact observation and experimental demonstration. In other words, we may now properly speak of medical science, for, while our knowledge in many directions is far from being complete, it is founded upon a scientific of observation and experiment, and is being rapidly extended by scientific methods."—*Sternberg.*

